# The similarity of the effect of carbohydrase or prebiotic supplementation in broilers aged 21 days, fed mixed cereal diets and challenged with coccidiosis infection

**DOI:** 10.1371/journal.pone.0229281

**Published:** 2020-02-24

**Authors:** Allison D. Craig, Farina Khattak, Peter Hastie, Mike R. Bedford, Oluyinka A. Olukosi

**Affiliations:** 1 Monogastric Science Research Centre, SRUC, Edinburgh, United Kingdom; 2 McCall Building, School of Veterinary Medicine, University of Glasgow, United Kingdom; 3 A B Vista, Woodstock Centre, Marlborough Business Park, Marlborough, United Kingdom; 4 Department of Poultry Science, University of Georgia, Athens, GA, United States of America; USDA-Agricultural Research Service, UNITED STATES

## Abstract

The aim of this study was to investigate the effect on growth performance and nutrient utilisation when supplementing diets deficient in energy and protein with carbohydrase enzymes or xylo-oligosaccharide in broilers challenged with coccidia. 960 Ross 308 broilers were used in this 21-day study. The treatments were arranged into a 2×4 factorial with 2 challenge states (challenged and non-challenged) and 4 different additive types (control, xylanase alone, xylanase and β-glucanase mixture and xylo-oligosaccharide). On day 14, the challenged group received 12× the recommended dose of coccidiosis vaccine while the non-challenged group received a sham treatment of water only. The birds and feed were weighed on days 0, 14 and 21. On day 21, two birds per pen were euthanized, the caeca were removed and the contents collected for short chain fatty acid analysis. Six more birds per pen were euthanized and ileal digesta were collected and pooled per pen for nutrient digestibility analysis. Feed intake was greater (P < 0.05) on days 14 and 21 when xylo-oligosaccharide was included in the diet compared to the xylanase and β-glucanase mixture in birds challenged with coccidiosis. Including xylo-oligosaccharide in the diet improved (P < 0.05) the digestibility of nitrogen and supplementing diets with the xylanase and β-glucanase mixture improved (P < 0.05) the digestibility of several amino acids. The concentration of arabinose and xylose was (P < 0.001) greater when broiler diets were supplemented with carbohydrase enzymes or xylo-oligosaccharide compared to the control. Although there was an increase in short chain fatty acid production due to the addition of carbohydrase enzymes or xylo-oligosaccharide, there was no additive effect on the %G+C profile of caecal bacteria however there was a negative effect of coccidiosis. In conclusion, the similarity in the response to carbohydrase enzymes or xylo-oligosaccharide supplementation illustrates that the hydrolysis products from carbohydrase activity may have prebiotic like effects.

## Introduction

Non-starch polysaccharide (NSP) is a group of large molecules found in cereal grains such as wheat and barley which are used in poultry diets and have negative effects on growth performance and nutrient utilisation caused by an increase in digesta viscosity [1]. In order to minimise these negative effects carbohydrase enzymes such as xylanase are added to cereal diets to hydrolyse NSP [2] and, in doing so, there is evidence to suggest that in-situ prebiotic oligosaccharides are generated [3–4].

Coccidiosis is a common disease in poultry which is characterised by gastrointestinal lesions and reduced growth performance [5] and is caused by several different Eimeria species [6]. Coccidiostats are commonly added to feed to inhibit the proliferation of Eimeria species that cause coccidiosis [7].

Carbohydrase enzymes have been shown to minimise the effects of coccidiosis [8] and necrotic enteritis [1], which are two common gastrointestinal diseases found in commercial poultry production systems. It has been suggested that the improvement in growth performance following enzyme addition was likely due to an increase in nutrient utilisation due to a decrease in ileal digesta viscosity [1].The literature also illustrates that supplementing broiler diets with prebiotic or carbohydrase enzymes alleviated the depression in body weight gain caused by sub-clinical coccidiosis [8].

The suggestion that prebiotic supplementation can enhance the immune response is based on the premise that prebiotics alter the gastrointestinal tract microflora by creating favourable conditions for beneficial bacteria to flourish while discouraging the proliferation of pathogenic bacteria [9]. It has been reported that supplementing broiler diets with mannan-oligosaccharide (MOS) resulted in a reduction in coccidiosis lesions caused by Eimeria species [10] due to improving immune function.

The similarity in the effect of prebiotic and carbohydrase supplementation on the performance of broilers infected with coccididia may indicate a similarity in the additives function. This would support the suggestion that prebiotic oligosaccharides are liberated from the structure of NSP during enzyme hydrolysis [11].

The aim of the current study was to investigate the effect of supplementing mixed cereal diets with carbohydrase enzymes or XOS on the growth performance, nutrient utilisation and composition of caecal microflora, by measuring SCFA concentration and %G+C profiling of the caeca content, in broilers challenged with coccidiosis infection.

## Materials and methods

### Animals, diet and housing

Nine hundred and sixty-six male Ross 308 broilers were used in this 21 day study. This study followed a randomised complete block design and was arranged into a 2×4 factorial arrangement. The factors were 2 challenge states (challenged with coccidia and non- challenged (without coccidia challenge)) and 4 additive types (control (no additive), xylanase alone, xylanase and β-glucanase or 0.025% XOS). The birds in the challenged group were given 12× the recommended dose of coccidiosis vaccine (Paracox 8) to induce a mild infection. All of the diets were deficient in energy by 1 MJ/kg (12 MJ/kg) and protein by 30g/kg CP and contained 500FTU/kg of phytase activity. One unit of phytase activity is defined as the amount of enzymes required to liberate 1μmol of phosphate per minuet at pH 5.5 and 37°C. A nutrient matrix was applied to the diets containing phytase. The diets were formulated to contain 11g/kg lysine, 8.36g/kg methionine and cysteine, 4.18g/kg methionine, 7.26g/kg threonine, 8.36g/kg valine, 11.44g/kg arginine and 1.76g/kg tryptophan. The carbohydrase mixture was made up of xylanase (16,000XU/kg) and β-glucanase (16,000U/kg). One unit of xylanase activity is defined as the amount of enzyme required to liberate 1nmol of reducing sugars from xylan using a standardised test. One unit of β glucanase activity is defined as the amount of enzyme required to liberate 1nmol of reducing sugars from β- glucan using a standardised test. The xylanase (Econase XT), β-glucanase (Econase GT) and the phytase (Quantum Blue 10G) were all supplied by AB Vista, Marlborough, UK. The XOS used in this study was purchased from Shandong Lifelong Bio-technology Co., China. The protocol was approved by the Animal Ethics Committee of the Scottish Agricultural College (SRUC). Titanium dioxide was included in each of the diets and to act as an indigestible marker to allow for nutrient digestibility analysis. The diet formulation is shown in Table 1.

**Table 1:** Diet formulation and calculated and analysed nutrient value of the control diet fed to broilers from days 0 to 21 and supplemented with carbohydrase enzymes or xylo-oligosaccharide.

Ingredient	Control (g/kg)
Wheat	244.9
Wheat Bran	144
Corn	100
Wheat germ	95
Barley	177.5
Soybean meal	161
Soya oil	30
Salt	3
Limestone	11
Dicalcium Phosphate	10
Lysine HCl	6
Methionine	2.5
Threonine	2.5
Valine	2.5
Vitamin & Mineral premix	5
TiO Marker	5
Total	1000.0
Calculated content	Control (%)
ME (Kcal/kg)	2890.12
Crude Protein	20.62
Calcium	0.76
Phosphorus	0.70
nPP	0.37
Sodium	0.14
Chloride	0.37
Analysed nutrient content	Control (%)
DM	87.8
Sodium	0.11
Chloride	0.36
Calcium	0.83
Phosphorus	0.61
Nitrogen	3.22

Note: Limestone contained 39.8% Ca; Dicalcium Phos contained 25.7% Ca and 17.5 P; The premix provided (units kg^-1^ diets): Vit A 16,000 iu; Vit D33,000 iu; Vit E 75 iu; Vit B1 3mg; Vit B2 10 mg; Vit B6 3mg; Vit B12 15 μg; Vit K3 5mg; Nicotinic acid 60mg; Pantothenic acid 14.5mg; Folic acid 1.5mg; Biotin 275 μg; Choline chloride 250 mg; Iron 20mg; Copper 10 mg; Manganese 100 mg; Cobalt 1 mg; Zinc 82mg; Iodine 1mg; Selenium 0.2mg; Molybdenum 0.5mg

Birds were housed 15 per pen and allocated into eight treatments, eight replicates per treatment and 64 pens in total. The treatment structure was as follows; Treatment 1- challenged with control diet; 2- non-challenged with control diet; 3- challenged with xylanase (16,000 XU/kg) alone; 4- non-challenged environment with xylanase (16,000 XU/kg) alone; 5- challenged with xylanase and β-glucanase; 6- non-challenged with xylanase and β-glucanase; 7- challenged with XOS (0.025%) and 8- non-challenged with XOS (0.025%).

On day 14, the birds in the challenge group were given 12× the recommended dose of coccidiosis vaccine by oral inoculation. Each bird in the challenged group received 6000 *E*. *acervulina* HP, 1200 *E*. *brunetti* HP, 2400 *E*. *maxima* CP, 1200 *E*. *maxima* MFP, 12000 *E*. *mitis* HP, 6000 *E*. *necatrix* HP, 1200 *E*. *praecox* HP and 6000 *E*. *tennela* HP live sporulated oocytes per dose. The birds in the non- challenged group were given distilled water also by oral inoculation.

On day 21, two birds per pen were euthanised by cervical dislocation. The birds were individually weighed and caecal contents were collected for SCFA analysis and %Guanine + Cytoine (G+C) microbiome profiling. On day 21, an additional 6 birds per pen were euthanised and the ileal digesta was collected and pooled per pen to calculate nutrient digestibility.

### Data collection

#### Growth performance

The birds and feed were weighed per pen on days 0, 14 and 21 to give body weight gain (BWG), feed intake (FI) and feed conversion ratio (FCR).

#### Nutrient utilisation and feed analysis

Ileal digesta was collected on day 21, freeze dried and analysed for dry matter (DM), amino acid content, titanium dioxide (Ti) and nitrogen (N). Samples of feed were taken on arrival to the farm and analysed for DM, N, Ti and all test products (xylanase, β- glucanse, phytase and the carbohydrate composition of XOS).

#### Oocyst counts

Fresh excreta were collected from each pen on day 21 and analysed for coccidial oocysts using the modified Mc Master counting chamber technique as describedin the literature [12].

#### Chemical analysis

The analyses of Ti in diet and digesta samples were carried out using a methodfound the in the literature [13]. DM was determined by drying 1g of the sample in a forced draft drying oven (Unitherm, Russel-Lidsay Engineering Ltd, Birmingham, England, UK) at 95° for 24 hours (Method 934.01; [14]). The N and amino acid contents of the diets and digesta samples were carried out using standard methods. N content was determined using the combustion method (Method 968.06; [14]) and amino acid content was determined using chromatography (Method 985.28; [14]).

The ileal digesta collected on day 21 was freeze dried and analysed for carbohydrate components of NSP by high performance liquid chromatography (HPLC) [15] The caecal content collected on day 21 was analysed for short chain fatty acid (SCFA) content following the method by Kettunen *et al*. (2015) 0.4g of caecal content was weighed out and added to 2.4ml of pivalic acid before being centrifuged. 800μl of the supernatant was then precipitated by adding 400μl of saturated oxalic acid solution before being incubated for 60min at 4°C. The samples were then centrifuged for the final time and analysed by gas liquid chromatography.

The caeca content collected on day 21 was analysed for the population of several different microbes using G+C profiling [16] The isolated bacteria were lysed and the DNA was purified using mechanical and chemical cell lysis [16]. Once the DNA was purified, it was split by CsCl equilibrium density gradient centrifugation which separates chromosomes based on their G+C content. Following separation of the chromosomes, the mixture was ultracentrifuged and passed through an ISCO UA-5 UV absorbance detector (ISCO Inc., Lincoln, Ne, USA) set at 280nm. The % G+C profile was generated using linear regression obtained from control samples with known % G+C composition [17].

#### Calculations

Nutrient digestibility was calculated using the following equations [18]):

$$Dry\;matter\;digestibility\;(DMD)=1-\left(\frac{Ti\;in\;diet}{Ti\;in\;digesta}\right)$$

$$Nutrient\;digestibility\;(\%)=\left[1-(\frac{Ti\;in\;diet}{Ti\;in\;digesta})\times (\frac{Nutrient\;in\;digesta}{Nutrient\;i\;ndiet})\right]\times 100$$

Pre-caecal NSP concentration (g/100g) was calculated using the equation below [19];

$$NSP\;Concentration=NSP\;conc.\;in\;digesta\times \left(\frac{Ti\;in\;diet}{Ti\;in\;digesta}\right)$$

#### Statistical analysis

The statistical analysis was carried out using Genstat 16^th^ Edition. Differences were found to be significant at P ≤ 0.05 or trends were identified at 0.05 > P < 0.1. The data were compared using the general ANOVA function assuming that the population is distributed normally. If the population was not normally distributed, the data were transformed prior to analysis. If a significant interaction was identified, the means were separated using a tukeys test.

## Results

The formulation and analysed nutrient composition of the control diet is shown in Table 1. The carbohydrate composition of all of the experimental diets and the XOS are displayed in Table 2.

**Table 2:** Analysed non-starch carbohydrate content (g/100g) of experimental diets 1–4 and the xylo-oligosaccharide fed to broilers for 28 days.

Carbohydrate fraction	Diet 1	Diet 2	Diet 3	Diet 4	XOS
Soluble NSP					
Rhamnose	0.00	0.00	0.00	0.00	0.00
Arabinose	0.26	0.27	0.35	0.20	0.40
Fructose	0.00	0.00	0.00	0.00	0.00
Xylose	0.67	0.51	0.96	0.40	1.40
Mannose	0.00	0.21	0.25	0.24	0.10
Galactose	0.43	0.52	0.45	0.40	1.30
Glucose	1.69	1.15	1.59	0.81	1.10
GlcA2	0.00	0.20	0.00	0.07	0.00
Total soluble NSP	2.93	2.83	3.45	2.07	4.20
Insoluble NSP					
Rhamnose	0.04	0.04	0.03	0.04	0.00
Arabinose	2.90	2.65	2.48	2.61	0.50
Fructose	0.08	0.06	0.06	0.05	0.10
Xylose	4.42	4.03	3.55	3.87	4.60
Mannose	0.37	0.20	0.10	0.10	0.20
Galactose	0.88	0.89	0.87	3.22	0.20
Glucose	3.39	3.09	2.83	3.22	13.70
GlcA2	0.34	0.31	0.27	0.28	0.00
Total insoluble NSP	12.42	11.26	10.20	11.06	19.30

NSP- non-starch polysaccharide

XOS- xylo-oligosaccharide was the prebiotic oligosaccharide used in diet 4

Diet 1- control

Diet 2- control plus 16,000BXU/kg xylanase

Diet 3- control plus 16,000BXU/kg xylanase and 16,000BXU/kg β-glucanase

Diet 4- control plus 0.025% XOS

### Oocyte counts from broilers aged 21 days, fed mixed cereal diets supplemented with carbohydrase enzymes or xylo- oligosaccharides and challenged with coccidiosis

Coccidial oocyte counts are shown in Table 3. There was no significant challenge state × additive type interaction for oocytes counts on day 21. The oocyte count was greater (P < 0.001) in those birds challenged with coccidiosis compared to those that were not. There was no significant effect of additive type on coccidial oocyte counts.

**Table 3:** Oocyte counts from fresh excreta of broilers fed mixed cereal diets supplemented with carbohydrase enzymes or xylo-oligosaccharide and challenged with a mild coccidiosis infection.

Challenge state	Additive	Oocysts/g
Means for main effect of challenge		
Challenged		20093
Non- challenged		115.0
	SD	14127
Means for main effect of additive		
	Control	11672
	Xylanase alone	9020
	Xylanase and β-glucanase	10319
	XOS	31646
	SD	10709
P value		
Challenge state		<0.001
Additive type		0.506

Notes: the data was log transformed prior to analysis; XOS- xylo-oligosaccharide

### Growth performance of broilers aged 14 and 21 days, fed mixed cereal diets supplemented with carbohydrase enzymes or xylo- oligosaccharides and challenged with coccidiosis

The growth performance data is shown in Table 4. On day 14 BWG increased (P < 0.01) when the carbohydrase mixture or XOS were present in the diet compared to the control treatment. FCR decreased (P < 0.05) when the carbohydrase mixture or XOS were included in the diet compared to the control treatment.

**Table 4:** The growth performance of broilers aged 14 and 21 days fed mixed cereal diets supplemented with carbohydrase enzymes or xylo-oligosaccharide and challenged with coccidiosis infection.

		Day 14			Day 21		
Challenge state	Additive	BWG (g/bird)	FI (g/bird)	FCR	BWG (g/bird)	FI (g/bird)	FCR
Challenged	Control				656	1119^a^^b^	1.72
	Xylanase alone				691	1117^a^^b^	1.62
	Xylanase and β-glucanase				691	1055^a^	1.53
	XOS				698	1164^b^	1.68
Non-challenged	Control				655	1097^a^^b^	1.67
	Xylanase alone				674	1152^b^	1.71
	Xylanase and β-glucanase				704	1159^b^	1.65
	XOS				717	1096^a^^b^	1.53
	SEM				16.2	29.6	0.05
Challenged		Challenge not yet applied			684	1114	1.64
Non-challenged					688	1126	1.64
	SEM				8.091	14.42	0.026
	Control	291^a^	465	1.60^b^	655^a^	1108	1.70
	Xylanase alone	304^a^^b^	478	1.58^a^^b^	683^a^^b^	1135	1.67
	Xylanase and β-glucanase	316^b^	469	1.49^a^	698^a^^b^	1107	1.59
	XOS	323^b^	481	1.49^a^	708^b^	1130	1.60
	SEM	5.1	5.8	0.02	11.4	21.0	0.04
P Values							
Challenge state					0.778	0.578	0.918
Additive type		<0.001	0.244	0.014	0.013	0.726	0.272
Challenge state × additive type					0.693	0.044	0.104

BWG- body weight gain; FI- feed intake; FCR- feed conversion ratio; XOS-xylo-oligosaccharide

^abc^ different superscripts within the same column and environment type indicates that the means are significantly different

On day 21 there was a challenge state × additive type interaction for feed intake. Those birds raised in the challenged environment and receiving XOS in the diet and those birds raised in the non-challenged environment and receiving xylanase alone or xylanase and β-glucanase had greater (P < 0.05) BWG than birds raised in the challenged environment and receiving xylanase and β-glucanase in the diet. BWGwas greater (P < 0.05) when XOS was included in the diet compared to the control treatment.

### Ileal nutrient digestibility of broilers aged 21 days, fed mixed cereal diets supplemented with carbohydrase enzymes or xylo- oligosaccharides and challenged with coccidiosis

**Fig 1:**
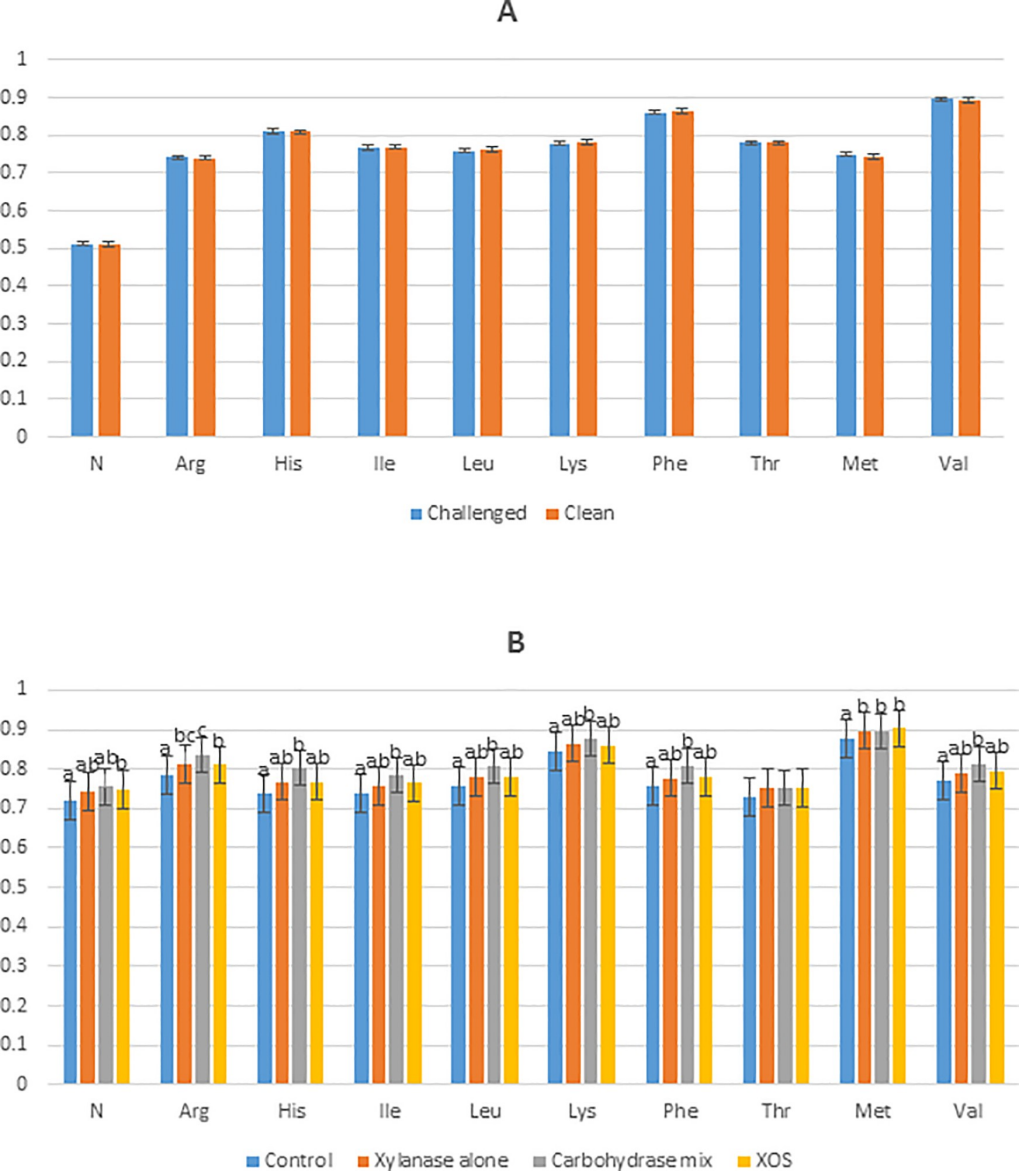
**The main effect of Challenge State (A) and Additive Type (B) on the coefficient of ileal digestibility of indispensable amino acids in broilers aged 21 days.** XOS-xylo-oligosaccharide; N- nitrogen, Arg- arginine; His- histidine; Ile- iso-leucine; Leu- leucine; Lys- lysine; Phe- phenylalanine; Thr- threonine; Met- methionine; Val- valine; ^abc^ different superscripts indicates that the means are significantly different.

The digestibility of N and indispensable amino acid were measured and some significant differences were identified (Fig 1). There was no significant challenge state × additive type interactions for N or indispensable amino acids however there were some effects of additive type. The digestibility of N was greater (P < 0.05) following XOS supplementation compared to the control treatment. The digestibility of arginine was greater (P < 0.001) following the supplementation of xylanase, or XOS compared to the control treatment and arginine digestibility was greater (P < 0.001) following xylanase and β-glucanase supplementation compared to XOS supplementation. The digestibility of histidine, isoleucine, leucine, phenylalanine, valine and lysine was greater (P < 0.05) following the supplementation of xylanase and β-glucanase compared to the control treatment. The digestibility of methionine was greater (P < 0.001) following xylanase, xylanase and β-glucanase or XOS supplementation compared to the control treatment.

### The ileal concentration of NSP fractions in broilers aged 21 days, fed mixed cereal diets supplemented with carbohydrase enzymes or xylo- oligosaccharides and challenged with coccidiosis

**Fig 2:**
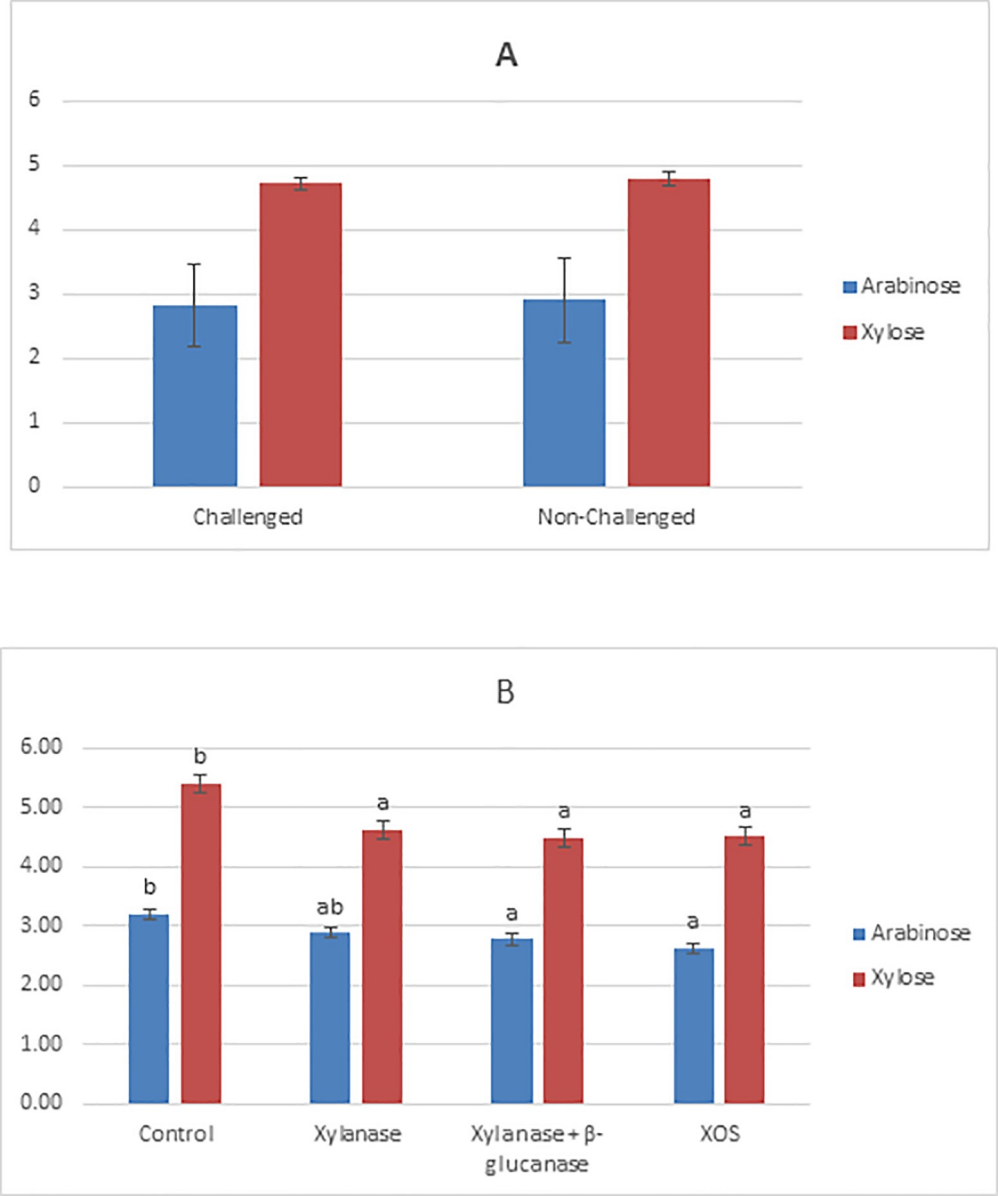
**The main effect of Challgene State (A) and Additive Type (B) on the ileal flow of insoluble arabinose and xylose from broilers aged 21 days.** ^ab^ superscripts show means that are significantly different.

The effect of coccidia challenge and carbohydrase or XOS supplementation on the ileal concentration of insoluble NSP was measured and some significant differences were identified (Fig 2). There was no challenge state × additive interaction effect for the ileal concentration of insoluble NSP. The supplementation of xylanase alone, the carbohydrase mixture or XOS reduced (P < 0.05) insoluble arabinose concentration compared to that of the control treatment. The supplementation of the carbohydrase mixture or XOS reduced (P < 0.001) insoluble xylose concentration. The supplementation of broiler diets with XOS reduced (P < 0.05) the ileal concentration of total insoluble NSP fractions compared to that of the control treatment. There was no significant effect of challenge state on the ileal concentration of insoluble NSP fractions.

### The concentration of SCFA in the caeca of broiler chickens aged 21 days and fed diets supplemented with carbohydrase enzyme or xylo-oligosaccharides and challenge with coccidiosis

The data for the proportion of SCFA in the caeca of broiler chickens aged 21 days is shown in Table 5. There was no challenge state × additive type interaction or challenge state effect in the current study however there was an effect of additive type. The percentage of propionic acid in the caeca was greater (P < 0.05) following XOS supplementation compared to the control treatment. There was a tendency (P = 0.081) for the percentage of lactic acid in the caeca to be lower following carbohydrase or XOS supplementation compared to the control treatment while the percentage of butyric acid tended (P = 0.096) to be greater following carbohydrase or XOS supplementation.

**Table 5:** The effect of supplementing mixed cereal diets with carbohydrase enzymes or xylo- oligosaccharide on SCFA concentration (mM) in the caecum of broilers challenged with coccidiosis infection.

Challenge State	Additive	Total SCFA	Lactic Acid	Butyric Acid	Propionic Acid	Acetic Acid	Valeric Acid
Means for the main effect of Challenge State							
Challenged		165.44	24.59	14.45	2.06	57.55	0.70
Non- challenged		156.02	23.80	14.71	1.83	58.37	0.64
	SEM	7.22	2.06	0.66	0.13	1.53	0.072
Means for the main effect of Additive Type							
	Control	176.49	28.32	13.28	1.63^a^	55.68	0.69
	Xylanase alone	163.13	27.30	13.76	1.68^a^^b^	56.23	0.50
	XY+BG	143.72	22.77	14.83	2.08^a^^b^	58.78	0.76
	XOS	159.59	18.40	16.45	2.39^b^	61.14	0.71
	SEM	10.22	2.92	0.930	0.190	2.16	0.101
P value							
Challenge state		0.363	0.790	0.779	0.223	0.708	0.587
Additive type		0.177	0.081	0.096	0.023	0.274	0.297

XY+BG- xylanase + β-glucanase; XOS- xylo-oligosaccharide

^ab^ Means in the same column, with different superscripts are significantly (P < 0.05) different

### The effect of coccidiosis challenge on the %G+C profile of the caecal microbiota in broilers fed mixed cereal diets supplemented with carbohydrases or xylo-oligosaccharide

There were no significant challenge state × additive type interactions for the %G+C profiles in the current study therefore the graph of the interaction effect is not presented.

**Fig 3:**
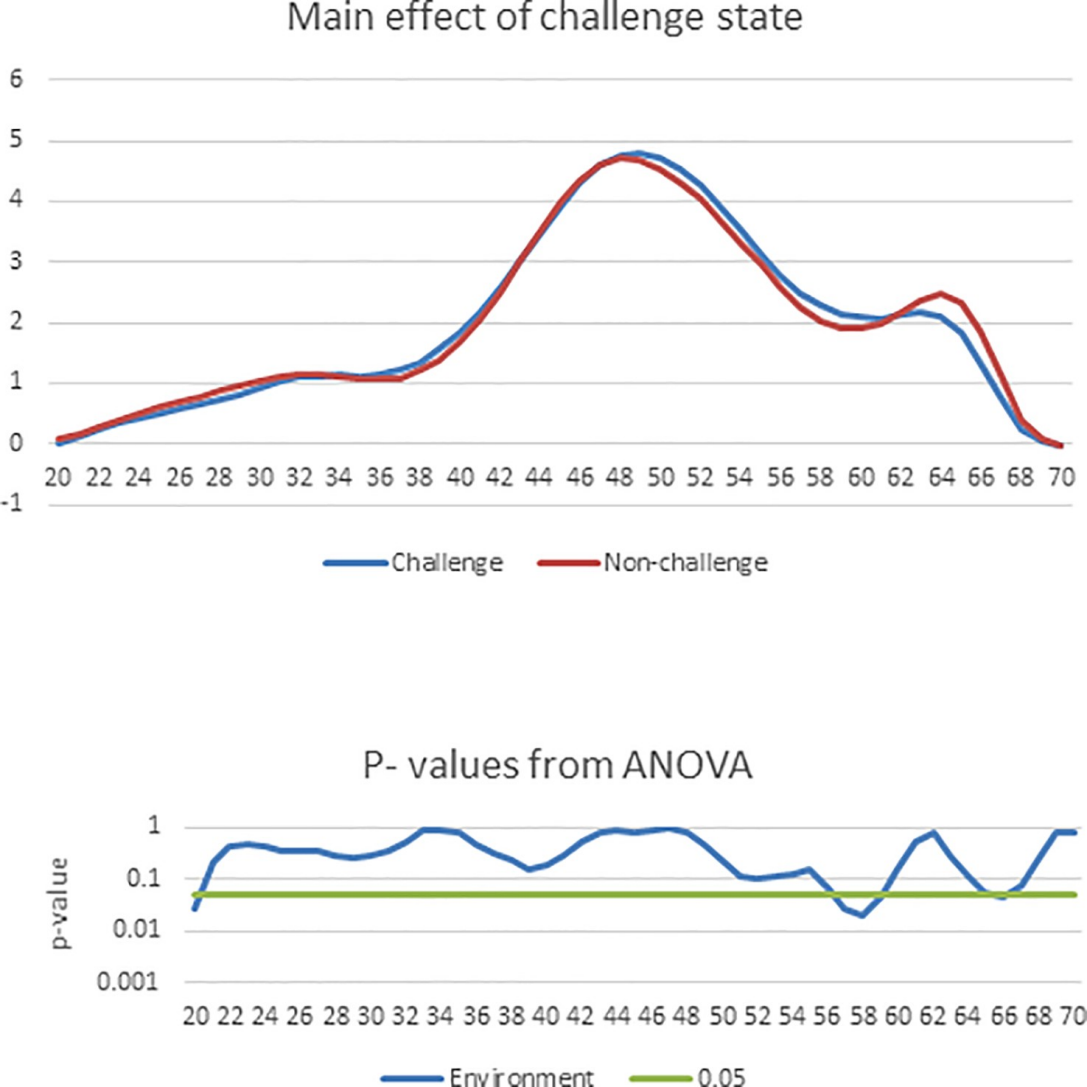
The effect of coccidiosis challenge on the %G+C profile of broilers aged 21 days. In the upper panel, the solid red line represents the mean %G+C profile of the non-challenged group and the solid blue line represents the mean %G+C profile of the challenged group. In the lower panel, the solid blue line shows the results from the ANOVA and the solid green line marks the threshold of P = 0.05.

Fig 3. shows the main effect of challenge state on the %G+C profile of caecal bacteria in birds aged 21 days. There was a significantly higher (P < 0.05) abundance of bacteria at %G+C 57–59 in the challenged group compared to the non-challenged group. At %G+C 66 there was a significantly higher (P< 0.05) abundance of bacteria in the non-challenged group compared with the challenge group.

**Fig 4:**
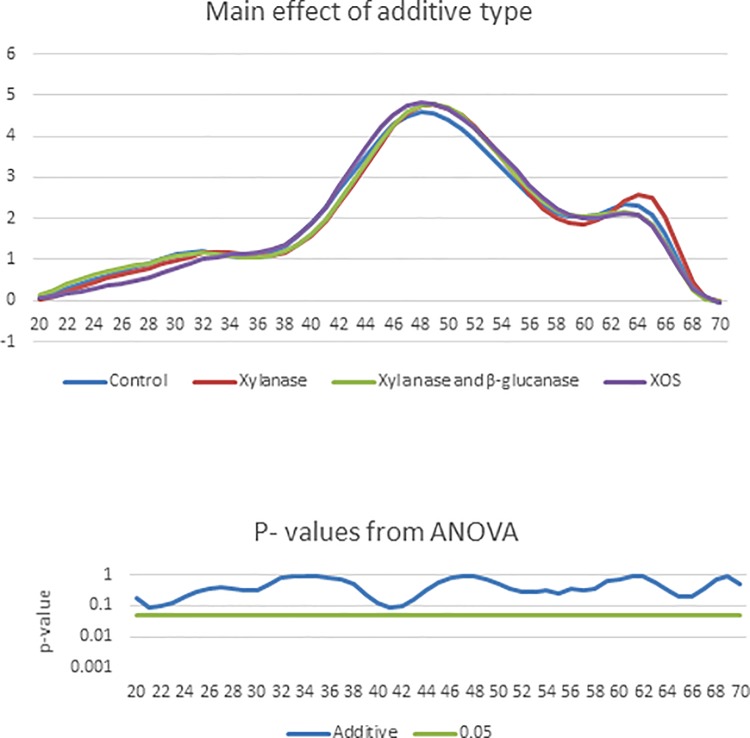
The effect of carbohydrase or xylo-oligosaccharide supplementation in broilers challenged with coccidiosis infection on %G+C profile of caecal microbiota. In the upper panel, the solid blue line represents the mean %G+C profile of the control group, the solid red line represents the mean %G+C profile of the xylanase group, the solid green line represents the mean %G+C profile of the xylanase and β-glucanase group and the solid purple line represents the mean %G+C profile of the XOS group. In the lower panel, the solid blue line shows the results from the ANOVA and the solid green line marks the threshold of P = 0.05.

Fig 4. shows the main effect of additive type on the %G+C profile of caecal bacteria in birds aged 21 days. There were no significant effects (P < 0.05) however there were some tendencies (P < 0.1). At %G+C 21–22, there tended (P = 0.090) to be a lower abundance of bacteria in the XOS group compared with the control, xylanase or xylanase and β- glucanase group. At %G+C 41, there tended (P = 0.092) to be a lower abundance of bacteria in the xylanase and β-glucanase group compared the XOS or control group.

## Discussion

Coccidiosis infection had no effect on growth performance, nutrient digestibility or in-soluble NSP concentration in the ileum of broilers aged 21 days. This could be due to the severity of the disease challenge. The aim of this disease challenge was to stress the birds’ immune system without causing a disease outbreak. Perhaps this level of coccidia challenge increased the amount of oocytes shed in the excreta but was not powerful enough to have a negative effect on bird performance.

The current study does show that the use of carbohydrase enzymes improves performance in birds at 14 and 21 days of age. BWG was improved when either the carbohydrase mixture or XOS were included in the diet. If the birds are healthy, the mechanism for improvements in performance are well established. Carbohydrase enzymes hydrolyse NSP reducing digesta viscosity. This enables the digesta to be properly mixed in the small intestine and increases nutrient absorption as well as releasing trapped nutrients from within the structure of NSP, increasing BWG [1,3]). This could be related to improvements in nutrient digestibility following xylanase and β-glucanase which has been investigated in the past and is in agreement with the literature [20, 21, 22]. In the current study, it is not possible to decide on a mechanism of action for the use of enzymes during coccidiosis infection as no data was recorded on intestinal lesion scores however, there were no significant differences in the number of oocytes collected from birds receiving different additives in the diet. This could suggest that the level of infection was similar among the different additives and the improvement in growth performance was not directly attributed to the damage to small intestine which is in agreement with the literature [8].

Prebiotic oligosaccharides are small molecules that are thought to have a beneficial effect on the microbial population in the small intestine. MOS has been shown to increase growth performance and reduce the severity of coccidiosis infection in broilers [23] however, other studies show only a reduction in the severity of coccidiosis lesions and no effect on growth performance [10]. The same can be said for β-glucan, it has been concluded that β-glucan reduced the severity of coccidiosis lesions however there was no significant effect on growth performance [24]. In the current study, XOS supplementation increased BWG and reduced FCR compared to that of the control treatment regardless of whether the birds were challenged with coccidiosis or not. In healthy birds, it is speculated that prebiotic oligosaccharides improve growth performance and nutrient digestibility by increasing the population of beneficial bacteria in the small intestine which, in turn, produce short chain fatty acids (SCFA)[25]. In birds challenged with *Eimeria*, supplementing the diet with a prebiotic will stimulate the immune system reducing the demand for energy, allowing the surplus energy to be used for growth, resulting in an improvement in performance [24].

Carbohydrase enzymes can also produce small oligosaccharides during the hydrolysis of NSP. The prebiotics produced by carbohydrase enzymes have shown similar effects on the integrity of the small intestine [26], which could contribute to the increase in nutrient utilisation following carbohydrase supplementation.

The concentration of insoluble NSP was reduced following the addition of carbohydrases or XOS in the current study. This is in agreement with previous studies that state that a reduction in the concentration of carbohydrates indicates an increase in bacterial fermentation in the intestine of broilers [22,27]. Recent publications have suggested an alternative mechanism of action for XOS whether the prebiotic is supplemented in the diet or results from NSP hydrolysis. It has been suggested that XOS encourages the bacteria in the intestine to digest xylan, found in the feed, more efficiently and consequently increases nutrient digestion overall contributing to the improvements in animal performance [28]. If the microflora is encouraged to digest xylan more efficiently, this would explain the reduction in insoluble xylose and arabinose concentration in the ileum of broilers following the addition of carbohydrases or XOS to the diet.

Given that supplementing broiler diets with carbohydrase enzymes or XOS both result in a similar reduction in insoluble xylan concentration, this supports the suggestion that the carbohydrate fractions resulting from the hydrolysis of NSP could act as prebiotic oligosaccharides.

The production of SCFA’s by the intestinal microflora is dependent on the types of bacteria present in the intestine and the types or amount of fermentable carbohydrates available [29]. Supplementing broiler diets with carbohydrases has been shown to increase SCFA production in other studies [30]. The mechanism behind this is thought to be through the production of prebiotic oligosaccharides resulting from NSP hydrolysis in the intestine [30]. In the current study the xylanase and β-glucanase mixture tended to have a greater effect on the proportion of lactic and butyric acid than supplementing diets with xylanase alone. Given that the diets used in the current study contained a mixture of cereals including wheat and barley, the use of more than one carbohydrase enzyme may have resulted in an increase in the diversity of fermentable carbohydrates resulting from enzyme hydrolysis. Although this is true for the percentage of lactic acid and butyric acid, the total amount of SCFA’s in the caeca did not differ between treatments raising doubt as it to the benefit of supplementing diets with more than one carbohydrase enzyme.

Given that enzyme hydrolysis results in a diverse range of carbohydrates, it is reasonable to suggest that enzyme supplementation may result in a greater production of SCFA compared to purified XOS supplementation. However, in the current study, this was not the case. Supplementing broiler diets with XOS had a greater effect on the proportion of different types of SCFA’s in the caeca than supplementing diets with carbohydrase enzymes. Although this was unexpected, it could indicate that while enzyme hydrolysis results in a diverse range of carbohydrates, the concentration of the carbohydrates may be lower than the concentration of purified XOS. This could explain why the addition of XOS had a greater impact on SCFA concentration than supplementing diets with carbohydrase enzymes.

The increased proportion of beneficial SCFA’s following carbohydrase or XOS supplementation in the current study could also be linked to the concentration of carbohydrate fractions in the ileum. The concentration of xylan and arabinose were reduced in the current study following the supplementation of carbohydrases or XOS while the proportion of propionic and butyric acid in the caecum tended to increase. Several different types of micro-organism have been shown to ferment XOS, xylan and arabinose such as *Lactobacilli*, *Clostridium species*, *Bifidobacterium* and *Bacteroides* [31]. Members of these bacterial species have also been shown to produce butyric acid or ferment larger molecules which can be used to produce SCFA [32]. Given that the fermentation of xylan and arabinose were increased and the production of butyric and propionic acid tended to increase, it is possible that the two outcomes are linked.

There was no significant effect of additive supplementation on the %G+C profile of caecal micro-organisms, however challenging birds with coccidiosis had a significant effect. It was interesting to see a significantly higher abundance of micro-organisms with %G+C 57–59 in the challenged group compared with the non-challenged group. This could represent *Bifidobacteria* [33] and thus could partly explain why no differences were seen in growth performance of challenged vs no challenged birds. This could very likely be because the composition of the intestinal microbiota at any point in time is dependent on several factors including the availability of nutrients and the immune status of the animal [34]. During coccidiosis infection the wall of the small intestine is damaged as part of the parasites lifecycle. This results in a decrease in nutrient absorption which can increase the amount of nutrients available to the microbiota allowing organisms, which may not flourish under normal circumstances, to replicate [35]. Bacterial species such as *E*.*coli*, *Salmoella* and *Enterobacter* have been shown to increase following *Eimeria* infection [36]. The reasons for this include changes in the passage rate of digesta, damage to the epithelium, an increase in gut pH and activation of the hosts’ immune system [36]. Although coccidiosis infection did cause a shift in the types of bacteria present in the caeca, there was no significant increase in %G+C range 20–30 which is often associated with pathogenic bacteria [16]

## Conclusion

The addition of carbohydrase enzymes or xylo-oligosaccharides had a positive effect on growth performance, nutrient digestibility, ileal NSP concentration and the proportion of SCFA. Although additive supplementation had no effect on the %G+C profile of caecal micro-organisms, the similarity in the effect of carbohydrases and XOS supplementation provides further evidence to suggest that products of NSP hydrolysis can act as prebiotics by altering the proportion of SCFAs in the caecum and merits further investigation.

## Supporting information

S1 Data File S1-S6 Tables data file. (XLSX)
